# Chemical forms of cadmium in soil and its distribution in French marigold sub-cells in response to chelator GLDA

**DOI:** 10.1038/s41598-022-20780-w

**Published:** 2022-10-20

**Authors:** Hongchuan Li, Deming Kong, Borui Zhang, Yusef Kianpoor Kalkhajeh, Yingying Zhao, Jieying Huang, Hongxiang Hu

**Affiliations:** 1 Anhui Province Key Lab of Farmland Ecological Conservation and Pollution Prevention, School of Resource and Environment, Anhui Agricultural University, Hefei, 230036 People’s Republic of China; 2 Department of Environmental Science, College of Science and Technology, Wenzhou-Kean University, 88 Daxue Road, Ouhai, Wenzhou, 325060 Zhejiang People’s Republic of China

**Keywords:** Ecology, Environmental sciences

## Abstract

The use of degradable chelating agents to facilitate phytoextraction is a promising low-cost method for the remediation of heavy metal-contaminated soils. However, there are few studies on how plants and soils respond to the chelating agents. In this study, the responses of French marigold (*Tagetes patula *L.) and soil cadmium (Cd) to the chelator tetrasodium glutamate (GLDA) was investigated in a 180 d field trial. Five GLDA treatments (0, 292.5, 585, 1170, and 2340 kg hm^−2^) were carried out in a Cd-contaminated soil (0.47 mg kg^−1^) under French marigold plantation. The results showed that the application of GLDA promoted the transformation of other forms of Cd in soil to exchangeable state, and the exchangeable Cd and Fe–Mn oxide bound state increased by 42.13% and 32.97% (*p* < 0.05), respectively. The cell wall Cd accumulations significantly increased 9.39% (*p* < 0.05) and the percentages of soluble fractions increased by 460.33% (*p* < 0.05). Furthermore, increases occurred in soil pH, as well as DOC and DTPA-Cd contents with increasing the total amount of GLDA. The composite application of GLDA (2340 kg hm^−2^) with French marigold reduced the total soil Cd content by 7.59% compared with the soil background. Altogether, results of this study suggested that the application of GLDA can effectively activate soil Cd and enhance the capability of French marigold for the remediation of Cd-contaminated soils.

## Introduction

Soil heavy metal pollution is a major ecological problem in urban areas^1^. Among which, cadmium (Cd) is one of the most mobile and biotoxic elements threating soils health^2^. If enters the plant in excess amounts, Cd can damage plant growth and development, inhibiting photosynthetic respiration of leaves and reducing their uptake of nutrient elements^3^. In human bodies, Cd can cause damages to the bones, immune system, and nervous system, resulting in serious risks of carcinogenesis, teratogenesis, and mutagenesis^4^. Therefore, it is of major importance to reduce the Cd content in the soil.

Chelator-enhanced plant uptake and accumulation of heavy metals is one of the most promising technologies for the remediation of heavy metal contaminated soils^5^. Chelating agents can form soluble complexes with insoluble heavy metals to facilitate their transfer from soil to the plants^6^. The organic chelator tetrasodium glutamate diacetate (GLDA) is a novel green and harmless chelator with sufficient biodegradability^7^. The degradation rate of GLDA can reach more than 60% within 28 days, and its degradation products have no adverse effects on the environment^8^. The drenching test showed that GLDA has a strong activation capacity for soil heavy metals, and it can be used to enhance plant uptake and accumulation of soil heavy metals^9^. Some hyperaccumulative plants can accumulate large amounts of Cd in their above ground parts without being affected by Cd toxicity; Although different genotypes of crops accumulate the identical amount of Cd in each organ, but the degree of damage varies greatly that, in turn, is closely connected to the subcellular distribution of intracellular Cd^10^. Subcellular localization of heavy metals can reveal the accumulation and tolerance mechanisms of plants to heavy metals^11–13^. Results of subcellular distribution of Cd in some woody plants suggested that cell walls and vesicles play important roles in the heavy metals’ tolerance process^14–17^. At present, only some indoor and potted experiments have been conducted to prove that GLDA has good potential for strengthening plants such as Southeast Jingtian and elephant grass^18,19^. However, most of the plants studied had low biomass and poor resistance to cadmium stress^20^. To our best knowledge of literature review, there are few studies addressing the subcellular distribution of Cd in French marigold. Likewise, the morphological transformation of soil heavy metals after the application of activators have received relatively less attention. Furthermore, the effect of actual agricultural soil remediation by GLDA may be significantly different from the potted trials due to runoff leaching and biodegradation.

Therefore, in this paper, GLDA was combined with French marigold to carry out a plot experiment for the remediation of a Cd-contaminated farmland soil. In this experiment, we investigated the responses of subcellular distribution of Cd fractions in French marigold, as well as soil DOC, and Cd morphology to the application of different doses of GLDA. We are hopeful that the results of this study can provide a methodological reference for the remediation of Cd-contaminated soils.

## Materials and methods

### Experimental materials and instruments

Site Description: The experimental site was selected around a small enterprise in Anhui Province, China, in a northern subtropical monsoon climate zone (Longitude: 117.17; Latitude: 32.48). In this area, the average annual temperature and precipitation are about 13 °C and 1100 mm, respectively. The topography is hilly-terrain and the soil type is Luvisols. Table 1 shows the basic physical and chemical properties of the study soils.

**Table 1:** Physical and chemical properties of the soil.

	pH	Total soil Cd mg·kg^−1^	DTPA-Cd mg·kg^−1^	Organic matter/g·kg^−1^	Alkali-hydrolyzable nitrogen/ mg·kg^−1^	Olsen-P mg·kg^−1^	DOC/mg·kg^−1^	Viscous particles /%
A disposal	6.53	0.47	0.09	30.71	253.06	53.51	82.98	23.42

#### Plant

Yellow variety of French marigold. Buy from Jiangsu Forest Tree Seed Industry Company.

#### Materials

Tetrasodium glutamate diacetate (GLDA) was purchased from Anhui Cool Biological Engineering Co. Sucrose; Tris–HCl, Dithiothreitol, MgCl_2_, NaAc-HAc, and NH_2_OH-HCl were purchased from Shanghai Aladdin Biochemical Technology Co. Other reagents (HCl, HNO_3_, HClO_4_, etc.) were guaranteed grade reagents, and could be used without further purification.

#### Instrument

Acidimeter (STARTER 3100) for soil pH; Coolable thermostatic shaker (IS-RDD3, USA) for temperature regulation and control during DTPA-Cd extraction; Graphite furnace-flame spectrophotometer (iCE 3500 Thermo, Thermo Fisher Scientific Ltd.) for the determination of Cd concentration; and TOC analyzer (TOC-V CPN FA, CN200, Shimadzu Corporation, Japan) for the determination of soil DOC.

### Experimental design

After rototilling the cultivated layer of the farmland, 15 plots with an individual area of 4 m^2^ (2 m × 2 m) were divided within the contaminated field. A ditch (20 cm width and 30 cm depth) was dug between the plots. To eliminate the surface runoff, plastic plates were inserted into ditches. For individual Cd contamination classes, five different treatments were practiced including control (CK), total GLDA application of 292.5 kg hm^−2^ (A1), total GLDA application of 585 kg hm^−2^ (A2), total GLDA application of 1170 kg hm^−2^ (A3), and total GLDA application of 2340 kg hm^−2^ (A4). In GLDA amended plots, the total GLDA was divided in two averaged applications with 15 days interval. In each plot, 12 French marigold seedlings were transplanted evenly on May 10, 2020. The first part of GLDA was applied after 60 days of cultivation, and the second application took place 15 days later. To do so, GLDA was dissolved in deionized water and uniformly applied to the inter-root soil with 25-L buckets. French marigold and soil samples were collected 180 days after seedlings’ transplantation. We applied identical fertilization scheme for all plots, i.e., compound fertilizer (25:10:16 N:P_2_O_5_:K_2_O) was applied simultaneously with seedlings’ transplantation at a rate of 200 kg hm^−2^, and urea (total N ≥ 46.0%) was added 30 days after seedlings’ transplantation at a rate of 1100 kg hm^−2^. Compound fertilizer and urea were applied by burrowing and spreading, respectively. All treatments were replicated three times.

### Sample collection and analysis

Determination of Cd content in each part: The above-ground and below-ground dry samples were weighed, 0.2 g each was digested by 10 mL HNO_3_-H_2_O_2_ in microwave for 5 h. After digestion and volume determination, Cd concentration was determined by graphite furnace-flame spectrophotometer via the quality control of national standard plant sample of celery (GBW 10048).

Subcellular distribution of Cd in French marigold: 0.50 g of the deionized water-washed fresh leaves was washed in a pre-chilled buffer [0.25 M sucrose, 1.0 mM dithioerythritol, and 50 mM Tris–HCl (pH 7.5)], and then grounded to homogenate to a final volume of 20 mL. All these steps were performed at 4 °C. The homogenates were centrifuged at 3000 r min^−1^ for 15 min, and the precipitated fraction obtained was the cell wall fraction (F1); the resulting supernatant was centrifuged at 15,000 r-min^−1^ for 30 min and separated again to obtain the organelle fraction (F2); the supernatant was the cell-soluble soluble fraction (F3). All fractions were extracted and digested with HNO_3_:HClO_4_ (9:1, v/v) as described above. A graphite furnace atomic absorption spectrophotometer (iCE 3500 Thermo, Thermo Fisher Scientific Ltd.) was used to determine the concentration of subcellular Cd.

Soil sample collection and analysis: Composite soil samples were collected to the depth of plant roots. Prior to the analyses, plant residues were removed, soil samples were air-dried and sieved through 1 mm and 0.149 mm, respectively, and then stored in self-sealing bags. Total amount of soil Cd was determined according to GB/T 17141-1997. Weigh 0.15 mm soil sample 0.2000 g, put it in the microwave effect tube, add 1 mL of HCLO_4_, 2 mL of HCL, 5 mL of HNO_3_. Efficient reaction was carried out at 160 °C. DTPA-Cd was determined according to HG 804-2016. Finally, the concentration was determined using a graphite furnace-flame spectrophotometer (iCE 3500 Thermo, Thermo Fisher). The detection limit was 0.02 μg·L^−1^, and each sample was measured three times, and then the quality control of the national standard sample (GSS-5) was used, and the recovery rate of Cd was 97.2–102.4%. DOC was determined with a TOC analyzer at a water-soil ratio of 5:1^21^. Cd morphology grading in soil: the five-step sequential extraction method proposed by Tessier et al. (1979) was used. This method partitions the heavy metals into five operationally defined chemical fractions: exchangeable, carbonate bound, iron and manganese oxides bound, organic matter bound, and residual.

### Data analysis

In this study, the correlation coefficients were calculated via the following equations:

1 $${\text{BCF}} = {\text{C}}_{{{\text{ds}}}} /{\text{C}}_{{\text{t}}}$$

2 $${\text{TI}} = {\text{C}}_{{{\text{ds}}}} /{\text{C}}_{{\text{g}}}$$

where BCF is the enrichment factor, indicating the ability of aboveground plant to enrich soil Cd; TI is the transfer factor, indicating the ability of aboveground plant to transfer Cd from the roots; C_ds_ is the Cd content (mg kg^−1^) of aboveground plant; C_t_ is the total Cd content in the soil (mg kg^−1^); and C_g_ is the root Cd content (mg kg^−1^).

The mean and standard deviation of the data were calculated using Excel 2010. The significance of differences, correlation analyses, and linear fitting were carried out using SPSS 20.0. The data were plotted using Origin 2017C.

## Results and discussion

### Effect of GLDA application on the biomass、enrichment and transfer of Cd in French marigold

The enrichment and transfer coefficients can reflect the enrichment and transfer characteristics of Cd in the soil-French marigold system^22^. As shown in Table 2, taking all treatments into account, French marigold biomass rises first and then declines, A3 treatment improved 32.26% compared to ACK. This is due to the low concentration of GLDA in promoting plant growth, while the high concentrations are inhibitory. The Cd contents of the upper and the lower parts of the peacock meadow were 0.19–0.37 and 0.54–0.84 mg kg^−1^, respectively. The BCF values of A1, A2, and A3 were all significantly higher than that of CK (*p* < 0.05), indicating that a small amount of GLDA application significantly improved the aboveground enrichment of soil Cd. However, there was no significant difference between A3 and A4, which may be due to the imbalance between the too fast DTPA-Cd translocation rate and the rate of DTPA-Cd uptake by French marigold itself, resulting in a blocked uptake of DTPA-Cd and inhibiting the significant accumulation of aboveground Cd with increasing total GLDA application^23^. The mean TI value of 0.43 was significantly higher than CK-TI of 0.35, indicating that GLDA application promoted the transfer of Cd from the roots to the aboveground^24^.

**Table 2:** Cd content and enrichment, and transport coefficient of maidenhair in different GLDA treatments.

	Biomass kg/m^2^	Cd content/mg kg^−1^		BCF	TI
		Above-ground plant parts	Foot end		
ACK	0.31 ± 0.03a	0.19 ± 0.02d	0.54 ± 0.01e	0.43 ± 0.03d	0.35 ± 0.02c
A1	0.32 ± 0.01a	0.24 ± 0.01c	0.63 ± 0.01d	0.54 ± 0.01c	0.38 ± 0.01bc
A2	0.35 ± 0.03a	0.31 ± 0.02b	0.64 ± 0.01c	0.71 ± 0.05b	0.48 ± 0.03ab
A3	0.41 ± 0.01a	0.35 ± 0.01ab	0.78 ± 0.01b	0.83 ± 0.03a	0.45 ± 0.02ab
A4	0.32 ± 0.02a	0.37 ± 0.02a	0.84 ± 0.01a	0.91 ± 0.03a	0.44 ± 0.01a

In the figure, there is a significant difference between different treatments for lower case letters (*p* ＜ 0.05), the same below.

### Effect of GLDA application on subcellular distribution of heavy metals in the upper part of French marigold

**Figure 1:**
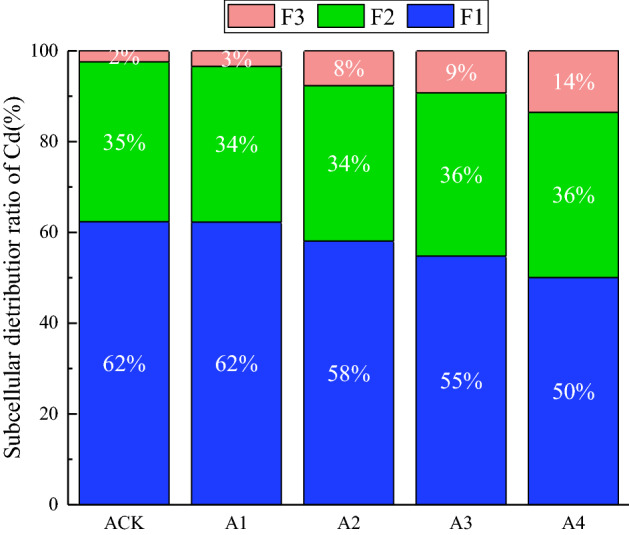
Subcellular distribution of Cd in maidenhair.

Effects of different rates of GLDA application on subcellular distribution of Cd in the upper part of French marigold are shown in Fig. 1. As can be seen, GLDA application significantly increased the Cd content in the cell wall (F1), organelles (F2), and soluble fractions (F3) of French marigold (*p* < 0.05) (Fig. 1). Correspondingly, the subcellular distribution of Cd fractions were ordered as cell wall (F1) > organelle (F2) > soluble fraction (F3). The percentages of cell walls and organelles decreased by 19.35% and 2.87%, respectively, whereas that of soluble fractions increased by 460.33%. The plant cell wall (F1) is the first site for Cd fixation after its transportation to the plant cells due to its proteins and polysaccharides components, adsorbing Cd ions and restricting their transport across the cell membrane^25,26^. Moreover, the surface of the plant cell wall is negatively charged and the Cd ion is positively charged, thereby facilitating Cd complexation^27^. The decrease in the proportion of Cd in the organelle (F2) may be due to the fact that the cell wall and empty vesicles sequester most of the Cd entering the plant, enhancing the compartmentalization of the cytoplasm and mitigating Cd damage to the cells, as well as maintaining the normal physiological and metabolic functions of the plant^28^. The soluble fraction (F3) mainly consists of vesicles, which contain many proteins, sugars, and organic acids. The latter can bind heavy metals to reduce their effectiveness and to decrease their transportation to the organelle^29,30^, thus reducing the stress of cadmium on plants. A significant increase occurred in the percentage of cellular soluble Cd content with increasing GLDA application, likely due to the high content of sulfur-rich states and organic acids in the vesicles that chelate and segregate Cd, thus eliminating Cd damage to the organelles^31^. The increase of Cd content in soluble fraction was flatten followed by a reduction, similar to Floodgate's principle^32^. Less Cd entered the cell at low DTPA-Cd concentrations, but when the Cd concentration exceeded a certain threshold, the Cd transport to the cell would suddenly increase, leading to an increase in the percentage of soluble fraction. It indicates that applying glda enhances the absorption of Cd in soil by French marigold.

### Effects of GLDA application on soil pH and DOC

**Figure 2:**
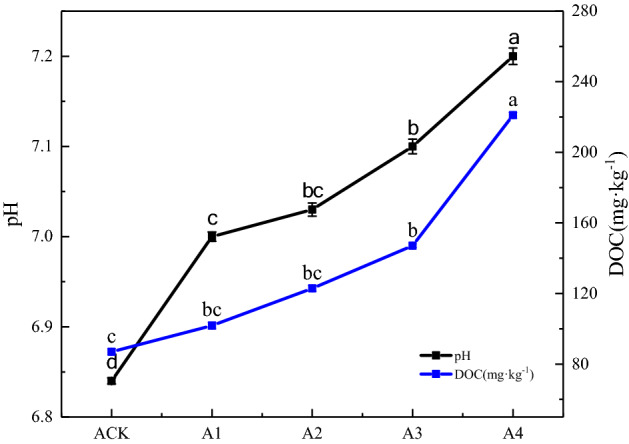
Soil pH and DOC in different GLDA treatments.

Soil pH is the main factor affecting the release of heavy metals^33,34^. Along with DOC, soil pH can affect plant growth and Cd bioavailability^35^. Figure 2 shows the soil pH of different treatments. Accordingly, GLDA application significantly increased soil pH from 6.84 in CK to 7.2; The average pH value of A1 to A4 was 7.08, which increased with increasing the total GLDA application, revealing a significant increase in soil pH after GLDA application. The hydrolysis process of -COO- content of GLDA is as follows: –COO– + H2O=–COOH + OH–, thereby increasing the OH- ions in soil solution and subsequently solution pH^36^.

From Fig. 2, the DOC mass concentration of CK was 86.94 mg kg^−1^, and those of A3 and A4 were 147 mg kg^−1^ and 221 mg kg^−1^, respectively, significantly higher than CK. This suggests that GLDA application significantly increased the mass concentration of soil DOC. The main source of DOC in soil systems is the decomposition of living organisms, and the increased DOC from the GLDA application may contain: soluble small molecule organic matter from GLDA degradation; GLDA on the activated portion of organic matter originally presents in the soil; stimulation of soluble organic acids and microbial secretion in French marigold roots by GLDA; soluble fraction of decomposition of other living organisms^37,38^. The GLDA degradation of DOC (up to 98% in 21d) was notably higher than that originally presents in the soil (generally not more than 2% of the total soil organic carbon) and the fraction that could be activated to DOC, suggesting that the increased DOC in this experiment was mainly from the GLDA degradation^39,40^. This shows that GLDA can improve the soil DOC content.

### Effects of GLDA application on soil total Cd and DTPA-Cd content

**Figure 3:**
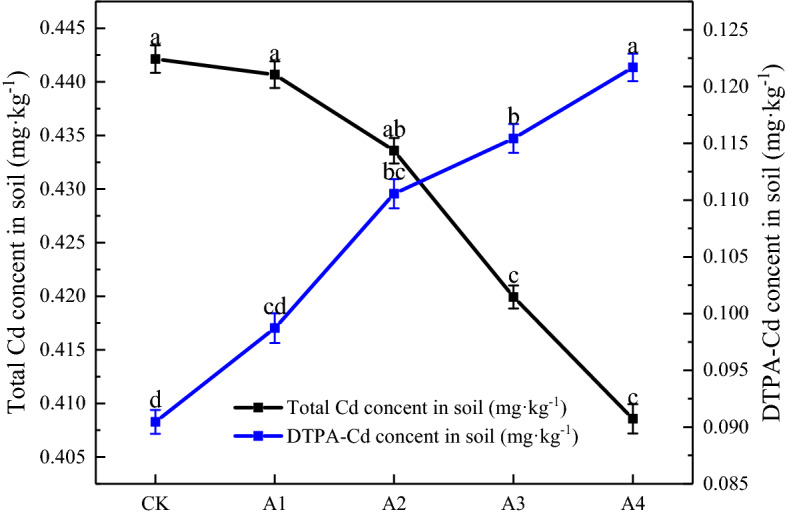
Soil total contents of Cd and DTPA-Cd in different GLDA treatments.

Chelators can induce the release of soil Cd and increase the DTPA-Cd content that, in turn, increases its enrichment by plants and decreases the total soil Cd content^41,42^. It can be seen from Fig. 3 that the soil total Cd content of each treatment decreased significantly with increasing GLDA application. The lowest soil total Cd content was 0.41 mg kg^−1^ under GLDA application rate of 2340 kg m^−2^, 7.59% lower than the CK.

GLDA application rate of 2340 kg m^−2^ significantly increased the effective Cd content by 35% compared with CK, while the effective Cd content did not change remarkably with increasing GLDA application (Fig. 3). This is in agreement with the results of Wang et al.^43^ who studied the effect of EDTA on Cd enrichment in foliar red beets. It can be seen that, due to the limitation of the background value of soil Cd at a certain concentration, large application GLDA can increase the soil DTPA-Cd content until it is stabilized.

### Effect of GLDA application on Cd morphology in soil

**Figure 4:**
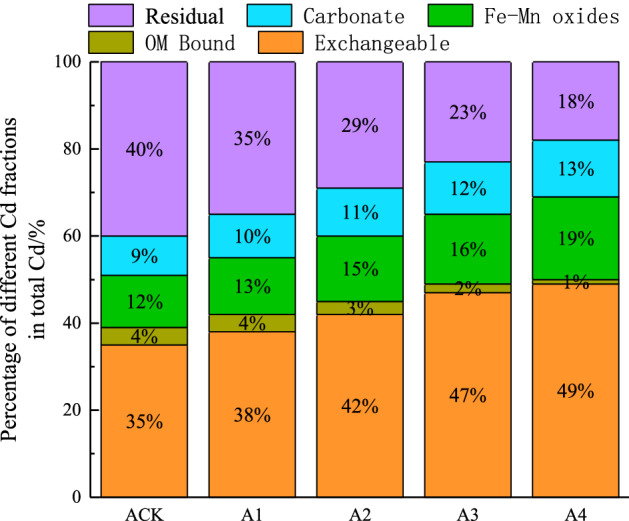
Effects of GLDA treatments on soil Cd fractions.

The fugitive morphology of heavy metals in soils directly affects their toxicity and environmental behavior^44^. Figure 4 shows that Cd in CK soils was mainly existed in the residual state, followed by the exchangeable state, Fe–Mn oxide bound state, and carbonate bound state, with the least availability in organic bound state; A1, A2, A3, and A4 treatments significantly increased the exchangeable Cd by 8.57%, 19.86% , 34.29%, and 42.13%, respectively, compared with CK; Fe–Mn oxide bound state increased by 32.97% under A4 treatment, and the residual and organic bound states both decreased to the different degrees by 31.34% and 74.84%, respectively. This indicates that GLDA application promoted the conversion of other forms of soil Cd into the exchangeable state, with the greatest decrease of 29.97% in the residual Cd state.

Exchangeable state is the most activated form of heavy metals, that is highly mobile and toxic, and directly plant accessible; residual state is the most stable form of heavy metals that cannot be removed and used by the plants; other forms are less active but undergo transformation under certain specific conditions^45,46^. Figure 4 also demonstrates that GLDA application changed the fugitive morphology of soil Cd, and facilitated its extraction by French marigold by increasing and decreasing the contents of exchangeable and residual Cd, respectively, thus increasing the effectiveness of Cd.

## Conclusion

1. GLDA application significantly increased the Cd content in the upper part of French marigold and promoted its transfer from the roots to the above ground. Besides, GLDA could increase the Cd content in the soluble part of the plant cells and promote cell wall fixation of Cd.
2. GLDA application significantly increased soil pH, as well as DOC and DTPA-Cd contents, while reducing soil total Cd content by 7.59% for 120 d of French marigold planting.
3. GLDA application significantly increased the contents of soil exchangeable and Fe–Mn oxide Cd, and reduced the contents of residual and organic bound Cd.
4. The less environmentally risky and biodegradable chelator GLDA has a major potential to enhance the phytoremediation of Cd-contaminated soils.

## Data Availability

The datasets used and/or analysed during the current study are available from the corresponding author on reasonable request.
